# The management of cancer in the elderly: targeted therapies in oncology

**DOI:** 10.1186/1742-4933-5-16

**Published:** 2008-12-30

**Authors:** Biagio Agostara, Giuseppe Carruba, Antonella Usset

**Affiliations:** 1 Clinical Oncology, Department of Oncology, "M. Ascoli" Cancer Hospital Center, ARNAS-Civico, Palermo, Italy; 2 Experimental Oncology, Department of Oncology, "M. Ascoli" Cancer Hospital Center, ARNAS-Civico, Palermo, Italy

## Abstract

Cancer is universally considered a disease of ageing. Today the management of elderly cancer patients poses many specific problems and it should be revisited in the light of the most recent advances in both diagnosis and treatment of human malignancies. In particular, the potential use of novel therapeutic options, based on therapeutic agents raised against molecular targets (the so called *targeted therapy*), appears to be promising in this clinical settings especially in view of the limited side-effects. The mainstays of cancer treatment during the twentieth century were surgery, radiation and chemotherapy. However, surgery is not curative in metastatic disease, radiation and chemotherapy are limited by side effects because they can't discriminate between healthy and cancerous cells. When key molecular changes responsible for malignant transformation were identified (e.g. growth factors and their receptors), it was hoped that new targeted agents, by inhibiting cancer-specific pathways, would spare normal cells and thereby offer improved safety benefits and a higher therapeutic index over standard chemotherapeutics. The most common targeted therapies used in clinical practice, i.e. monoclonal antibodies and small molecules, are described.

## Introduction: the ageing and cancer interface

Cancer is generally considered a disease of ageing, although the shared mechanisms underpinning the two processes remain unclear. Doubtlessly, incidence and mortality rates of most human cancers increase consistently with age, but they decline in the oldest old (≥ 90 years). This finding has been interpreted as a consequence of either the exploitation of large-scale population screening programs or the improvement of diagnostic capacities worldwide. However, there is convincing evidence that, regardless of other variables, cancer and aging remain associated until around 85 years of age, while they diverge substantially thereafter.

Recent studies have suggested that either "convergent" or "divergent" mechanisms may connect cancer and aging. In the former, molecular pathways simultaneously provide protection against cancer and resistance to ageing by limiting the generation and accumulation of cellular, genetic or epigenetic, damage. In the latter, some other pathways may eventually lead to protect from cancer but also to promote ageing, including shortening of telomeres and derepression of the *INK4a*/*ARF *locus.

It ought to be emphasized here that, by the year 2030, most patients with cancer will be aged over 65 years and many will be frail. Frailty implies a diminished physiologic reserve; contributors include diminished organ function, co-morbidities, impaired physical function, and geriatric syndromes. Oncologists are challenged to appropriately identify frail elderly cancer patients aiming to select appropriate therapies that will minimize toxicity and maintain quality of life. In this framework, the advent of targeted therapies and their potential use in the elderly clinical cancer setting is awaited with hope and growing interest.

The mainstays of cancer treatment during the twentieth century were surgery, radiation and chemotherapy. However, surgery is not curative in cases of metastatic disease, and radiation and chemotherapy are limited by severe side effects and a limited capacity to discriminate between healthy and cancerous cells.

In the 1980s, several groups began to identify the key molecular changes responsible for malignant transformation. These changes included the discovery of both cancer-causing oncogenes and tumor-suppressor genes that normally hold cancer in check, and the identification of epigenetic events such as promoter methylation that predispose individuals to cancer by switching genes that regulate cell growth on or off.

Cancer cells may acquire the capacity for autonomous and dysregulated proliferation through the uncontrolled production of specific molecules, generally encoded by oncogenes, that promote cell growth (growth factors) or through abnormal, enhanced expression of specific proteins (growth factor receptors) on the cell membranes to which growth factors selectively bind. Both processes trigger a series of intracellular signals that ultimately lead to the proliferation of cancer cells, induction of angiogenesis and metastasis. It was hoped that new targeted agents, by inhibiting cancer-specific pathways, would spare normal cells and thereby offer improved safety benefits over standard chemotherapeutics, while also providing a higher therapeutic index.

## Molecular targets and specific bullets: current clinical use and future perspectives

The human epidermal growth factor receptor (HER) family consists in four receptor proteins, that are transmembrane tyrosine kinases and bind to specific ligands: HER1 or epidermal growth factor receptor (EGFR), HER2, HER3 and HER4.

These receptors can be inhibited by either monoclonal antibodies (e.g. trastuzumab, cetuximab), that bind to the extracellular domain of the receptor when it is in the inactive configuration, compete for the receptor binding by occluding the ligand-binding region, and thereby block ligand-induced receptor associated tyrosine kinase activation, or by a tyrosine kinase inhibitor or small molecule (e.g. erlotinib, gefitinib, lapatinib), that competes with ATP to bind to the intracellular catalytic domain of receptor tyrosine kinase, thus inhibiting receptor autophosphorylation and downstream signaling.

HER2 normally regulates cell proliferation. Some tumours, in particular breast tumours, overepress HER2, a transmembrane receptor protein encoded by the proto-oncogene HER2/neu: infact, overexpression of HER2 (excess production of protein), amplification of the gene (excess number of copies) or both occur in approximately 20–30% of primary breast cancers, and are associated with a clinically aggressive tumour and a poor prognosis.

Overexpression of HER2 is detected by immunohistochemistry (IHC), the most frequently used method, while the amplification of the gene in the nuclei of affected cells can be detected using fluorescence in situ hybridization (FISH).

Trastuzumab was developed to target HER2: it's a recombinant, humanized IgG monoclonal antibody that selectively binds with high affinity to the extracellular domain of HER2 and inhibits the proliferation of tumour cells that overexpress HER2. The underlying mechanism of the antitumour effects of Trastuzumab has not been fully elucidated, but it may involve a number of different actions including: a) recruiting immune cells to target tumours through antibody-dependent cellular cytotoxicity (ADCC); b) inhibiting metalloproteinase-induced cleavage of the extracellular domain of HER2, thereby preventing the formation of a truncated HER2, which is thought to have a role in the aggressiveness of breast tumours; c) inhibiting HER2-mediated cellular proliferation that depends on the signaling transduction pathways P13K/Akt and MAPK, which are activated by HER-based dimers and are essential for cellular proliferation and survival; d) inhibiting HER2-mediated neo-angiogenesis.

Additive and/or synergistic effects have been demonstrated with trastuzumab and various cytotoxic drugs (paclitaxel, docetaxel, anthracyclines, cisplatin, carboplatin, vinorelbine, capecitabine) in HER2-overexpressing breast cancer cell lines and/or human breast xenograft models. These evidences represent the rationale for the concomitant use of this monoclonal antibody and chemotherapy.

The addition of intravenous trastuzumab to first-line chemotherapy improved the time to disease progression, objective response rate, duration of response, and overall survival in randomized, multicentre trials in women with HER2-positive metastatic breast cancer [1-11]. Thus, trastuzumab has become the standard of care in this setting.

Recent data from large phase III trials with trastuzumab in the adjuvant setting revealed significant improvements in disease-free and overall survival: trastuzumab has also become a component of adjuvant therapy for patients with HER2-positive early breast cancer [12-16]. Trastuzumab is under investigation in the neoadjuvant setting [17,18].

The most common adverse events associated with trastuzumab are infusion-related symptoms, such as fever and chills, usually occurring during the infusion of the first dose; this drug is also associated with a number of serious adverse events including cardiac events, severe hypersensitivity reactions (including anaphylaxis) and pulmonary events (infiltrates, pneumonia, fibrosis, pleural effusion, acute pulmonary oedema). In particular, the risk of ventricular dysfunction and congestive heart failure is particularly increased if trastuzumab is administered in combination with anthracycline-containing chemotherapy. The mechanism involved in trastuzumab-associated cardiac events is different from that associated with anthracycline: trastuzumab can activate HER2 receptor tyrosine kinases in human myocardium, despite their low level expression, resulting in cardiomyocyte disfunction. Patients should undergo a cardiac assessment prior to beginning trastuzumab therapy and cardiac function should be monitored during trastuzumab therapy.

HER2 is also overexpressed in other malignancies, including non-small cell lung cancer, gastric cancer, pancreatic cancer, but trastuzumab, in these tumours, did not show any clinical advantage.

Cetuximab is a human-mouse chimeric monoclonal antibody (IgG1 subtype) that binds to the extracellular domain of EGFR/HER1 and block ligand binding and receptor activation. Normally, after a ligand binds to a single-chain EGFR, the receptor forms a dimer that signals within the cell by activating receptor autophosphorylation through tyrosine kinase activity. Autophosphorylation triggers a series of intracellular pathways that may result in cancer cell proliferation, blocking apoptosis, activating invasion and metastasis, and stimulating tumor-induced neovascularization. This pathway is blocked by anti-EGFR drugs.

EGFR is expressed in the majority of human epithelial cancers and was the first growth factor receptor to be proposed as a target for cancer therapy. EGFR expression is usually determined by immunohistochemical methods and was the first biomarker investigated as a potential predictor of response to anti-EGFR drugs: however, most studies have failed to show any relationship between EGFR expression and the clinical activity of these drugs. Thus, in clinical practice Cetuximab is often administered in patients affected by tumors that do not overexpress this receptor.

A multicenter, randomized phase II trial evaluating the activity of cetuximab alone or with irinotecan in patients who did not respond to irinotecan therapy showed that the combination was more effective in terms of response rate and rate of progression-free survival, while the median survival was similar with the two approaches [19]. Other studies have investigated the drug in association with oxaliplatin +/- fluoropyrimidines (CRYSTAL, EXPLORE, COIN, INT N0147) and with Bevacizumab (20, CALGB 80405) in first and second line treatment for metastatic disease.

On the basis of these results, cetuximab has been approved in combination with irinotecan for the treatment of metastatic colorectal cancer that is refractory to irinotecan-based chemotherapy.

Cetuximab is also administered in combination with radiotherapy in patients with locally advanced squamous-cell carcinoma of the head and neck: infact, in a randomized phase III trial, radiotherapy and cetuximab significantly prolonged progression-free survival, duration of locoregional control and overall survival [21]. Other trials are investigating the drug in metastatic squamous-cell carcinoma of the head and neck in combination with the standard chemotherapy, cisplatin and fluorouracil [22,23]. Cetuximab will be soon approved in other malignancies, in particular NSCLC [24] and pancreatic cancer.

Dose-dependent and reversible diarrhea and acneiform rashes have been the most prominent side effects, which are very similar to those associated with the use of other tyrosine kinase inhibitors: a significant positive correlation between cutaneous toxicity and rates of response, progression-free survival and overall survival has been noted in virtually all trials.

Angiogenesis is a critical step in the growth and metastatic spread of solid tumours: infact, in the absence of angiogenesis, nascent tumors remain largely dormant, with a maximum diameter of 100 to 200 μm, owing to limitations in the diffusion of oxygen, glucose and waste products. Vascular endothelial growth factor (VEGF) has emerged as a key mediator of tumor angiogenesis and has recently been recognized as a potential target for therapy of solid tumours. The VEGF family comprises glycoproteins designated VEGF-A, VEGF-B, VEGF-C, VEGF-D and placental growth factor (PlGF): the best characterized of the VEGF family members is VEGF-A, a homodimeric glycoprotein that is expressed in four different isoforms and binds to VEGFR1 and VEGFR2.

Bevacizumab derives from a murine monoclonal anti-VEGF antibody, which is humanized (93% human) by incorporating murine VEGF-binding residues into human IgG framework. Bevacizumab causes inhibition of VEGF-induced proliferation, migration and survival of vascular endothelial cells and increases the permeability of these cells by preventing the binding of soluble VEGF to its receptors on the surface of endothelial cells. In addition to its direct antiangiogenetic effects, bevacizumab may also improve the delivery of chemotherapy by altering tumor vasculature, decreasing the elevated interstitial pressure in tumors and increasing the permeability of vascular endothelium.

On the basis of the results of a randomized trial demonstrating that the addition of bevacizumab to fluorouracil and leucovorin+irinotecan results in statistically significant and clinically meaningful improvement in survival among patients with previously untreated metastatic colorectal cancer [25], this drug has been approved for the treatment of these patients.

Bevacizumab is also approved in association with oxaliplatin-based regimens for first line treatment of metastatic colorectal cancer [26-28].

The drug has been approved for first line treatment of metastatic breast cancer in combination with paclitaxel [29], metastatic renal cancer in combination with interferon [30], and will be soon used for the treatment of NSCLC [31,32] and pancreatic cancer.

Potential side effects of bevacizumab include hypertension, proteinuria, bleeding and, infrequently, thrombotic events: life threatening events have occurred in a small number of patients.

The category of small molecules tyrosine kinase inhibitors includes erlotinib, lapatinib, sunitinib and sorafenib, all used in clinical practice.

Erlotinib is an oral EGFR tyrosine kinase inhibitor recently approved for the treatment of locally advanced and metastatic NSCLC patients who progress following 1 or 2 prior chemotherapy regimens. Its clinical use derives from the results of a phase III, randomized, double blind, placebo controlled study in which erlotinib increased median survival by approximately 2 months as compared with placebo. Response rates were significantly more frequent in women, in patients with adenocarcinoma, and in patients with no history of smoking: however, a significant survival advantage was observed in all patient subgroups [33].

On the basis of preclinical data demonstrating that anti-EGFR drugs potentiate the antitumor activity of cytotoxic drugs, randomized trials have examined the combination of erlotinib with chemotherapy as first-line treatment for NSCLC: neither a survival advantage nor a benefit with respect to the response rate or time to progression have been seen with the addition of erlotinib to chemotherapy in any of these trials [34,35].

Lapatinib is an oral ATP-competitive EGFR/HER1 and HER2 dual tyrosine kinase inhibitor: it inhibits receptor autophosphorylation and activation by binding to the ATP-binding pocket of the EGFR/HER2. In patients with HER2-positive advanced metastatic breast cancer that has progressed following treatment with previous chemotherapy regimens that included an anthracycline, a taxane, and trastuzumab, a randomized clinical trial has demonstrated that the addition of lapatinib to the chemotherapy drug capecitabine delayed the time of further cancer growth compared to capecitabine alone [36-38]. On the basis of these results, lapatinib was approved in United States in March 2007, for use in patients with advanced metastatic breast cancer in conjunction with capecitabine: it will be soon available in clinical practice also in our country.

Sunitinib and sorafenib are two orally administered multi-targeted tyrosine kinase inhibitors that have recently been approved for the treatment of metastatic renal-cell carcinoma (clear-cell histotype). Since renal-cell carcinoma is highly resistant to chemotherapy, interleukin-2 or interferon alfa were widely used as first line treatment of metastatic disease. Response rate with these cytokines were low (5 to 20%) and median overall survival was approximately 12 months. Alternative treatments have been lacking for renal-cell carcinoma since the development of these two innovative drugs.

Sunitinib inhibits tyrosine kinase associated with VEGFR2, PDGFRβ, with less potent activity against fibroblast growth factor receptor 1 (FGFR1) tyrosine kinase; sorafenib is an inhibitor of Raf-1, a member of the RAF/MEK/ERK signaling pathway, and of tyrosine kinase associated to VEGFR1, VEGFR2, VEGFR3, PDGFRβ, Flt-3 and c-KIT.

These receptor tyrosine kinases play a key role in the pathogenesis of clear-cell carcinoma, through involvement of the von Hippel-Lindau (VHL) gene, which is inactivated in up to 80% of sporadic cases of clear-cell carcinoma by deletion, mutation, or methylation. This tumor-suppressor gene encodes a protein that is involved in the regulation of the production of VEGF, PDGF and a number of other hypoxia-inducible proteins: inactivation of the VHL gene causes overexpression of these agonists of VEGFR and PDGFR and the resulting persistent stimulation of these receptors may promote tumor angiogenesis, tumor growth and metastasis. This is the rationale for the use of these small molecules in this tumor.

Sunitinib is approved for the first line treatment of metastatic clear-cell renal carcinoma after the publication of the results of a randomized phase III trial that has showed a longer progression-free survival and higher response rates, in the absence of survival benefit at the moment, in patients who received this drug than in those receiving interferon alfa [39]. Sunitinib is also approved for the second line treatment of gastrointestinal stromal tumors (GIST) [40] in progression after prior first line treatment with Imatinib [41], a multitarget tyrosine kinase inhibitor that principally blocks BCR-ABL kinase, c-kit and PDGFR-associated kinases.

Sorafenib is approved for the second line treatment of patients with metastatic clear-cell renal carcinoma in whom previous standard therapy has failed; this indication in clinical setting derives from the results of a randomized phase III trial that has showed longer progression-free survival with sorafenib as compared with placebo [42]. Sorafenib is also administered in patients with metastatic hepatocellular carcinoma [43], on the basis of the results of a phase III, double blind, placebo-controlled trial demonstrating that median survival and time to radiologic progression were nearly 3 months longer for patients treated with sorafenib than for those given placebo.

Both sunitinib and sorafenib are under investigation in other malignancies (NSCLC, melanoma, breast cancer, thyroid carcinoma, hormone-refractory prostate cancer, etc).

The most common side effects associated with these drugs are: fatigue, rash/desquamation, hand-foot skin reactions, diarrhea, hypertension, anaemia, neutropenia, other cardiac toxicities.

The drugs reported above represent only a part of the new category of biological targeted agents: many novel drugs are under investigation and will soon help oncologists in the fight against cancer (e.g. other tyrosine kinase inhibitors-ZD6474; other monoclonal antibodies-panitumumab, matuzumab, pertuzumab; m-TOR inhibitors-Temsirolimus, Everolimus; inhibitors of 26 S proteosome-bortezomib; histone deacetylase inhibitors-vorinostat; metalloproteinase inhibitors-marimastat, batimastat; etc).

There is an increasing interest in the use of target therapies in patients older than 65 years, which represent the fastest growing segment of the cancer population. Concerns about cancer treatment in the elderly relate to comorbidities, which increase proportionally with age, and physiological changes associated with aging, which may influence drug metabolism and toxicity. On the basis of subgroups analysis, the major published clinical trials suggest that administration of biological agents in this class of patients is effective and safe. It would be worthwhile to design clinical trials having the elderly as target population in order to better define the clinical use of these drugs in this subset of patients.

## Conclusion

Collaboration among laboratory scientists and clinical scientists hold tremendous promise. Three of the main future directions in the clinical use of target therapies are: 1) appropriate selection of patients who would benefit from treatment with these new drugs by means of molecular predictors of response; 2) determination of the most effective sequence and combination of biological drugs to use with chemotherapy, radiotherapy or both in order to optimize cytotoxicity potentiation; 3) introduction of biological drugs in the adjuvant setting, on the basis of the promising results obtained in the metastatic disease in order to improve outcomes; 4) increasing need to include the elderly population in clinical trials in order to define the possible role of biological drugs in this category of patients.

## Competing interests

The authors declare that they have no competing interests.

## Authors' contributions

BA, GC, and AU contributed equally to this paper. BA designed the review, GC and AU drafted the manuscript. All authors read and approved the final manuscript.
